# Open access chemical probes for epigenetic targets

**DOI:** 10.4155/fmc.15.127

**Published:** 2015-09-23

**Authors:** Peter J Brown, Susanne Müller

**Affiliations:** 1 Structural Genomics Consortium, University of Toronto, 101 College Street, Toronto, ON M5G 1L7, Canada; 2 Structural Genomics Consortium, University of Oxford, NDM Research Building, Roosevelt Drive, Oxford, OX3 7FZ, UK

## Abstract

**Background:**

High attrition rates in drug discovery call for new approaches to improve target validation. Academia is filling gaps, but often lacks the experience and resources of the pharmaceutical industry resulting in poorly characterized tool compounds.

**Discussion:**

The SGC has established an open access chemical probe consortium, currently encompassing ten pharmaceutical companies. One of its mandates is to create well-characterized inhibitors (chemical probes) for epigenetic targets to enable new biology and target validation for drug development.

**Conclusion:**

Epigenetic probe compounds have proven to be very valuable and have not only spurred a plethora of novel biological findings, but also provided starting points for clinical trials. These probes have proven to be critical complementation to traditional genetic targeting strategies and provided sometimes surprising results.

The term epigenetics was coined by Conrad Waddington in 1942, providing us with the memorable picture of the epigenetic landscape. In his famous picture of marbles running down a grooved hill toward a wall, he combined views of genetics and developmental biology. The marble at the top of the hill represents the pluripotent, undifferentiated stem cell, which through making different choices on its way downhill ends against the wall as a fully differentiated cell. The ‘choices’ in the shape of troughs are determined by the genetic and epigenetic set-up of the cell at a given time point and environment. However, Waddington’s definition did not provide an explanation as to the mechanisms of how epigenetic phenomena are regulated. Research in epigenetics originally focused on DNA modifications, in particular methylation, which was first suggested in 1969 to play a defining role in long-term memory. With the advent of new techniques to identify DNA modifications and the Epigenome project, much progress has been made to determine the pattern of cytosine methylation in a variety of cell types making DNA methylation one of the most extensively studied epigenetic marks [1]. Targeting these epigenetic modifications has been successful and in particular nucleotide analogs like 5-azacytidine (Aza) and 5-aza-2′-deoxycytidine (Aza-dC) have proven successful in a variety of cancers [2].

More recently, additional mechanisms are being explored including the role of regulatory RNAs like microRNAs (miRNAs), small noncoding RNAs of 20–24 nucleotides and long noncoding RNAs (lncRNAs) of up to 200 nucleotides [3,4]. Also, microRNAs have been shown to be amenable to small molecule intervention and the antibiotic streptomycin has been shown to inhibit miR-21 maturation by binding directly to the precursor of this microRNA [5].

Recent efforts generating small molecule inhibitors targeting histone tail modifications have been highly promising in terms of applied research. These post-translational modifications include most prominently methylation, acetylation and phosphorylation, but less frequent additional modifications such as crotonylation and citrullination are also being explored, and constitute a complex histone code [6]. Enzymes adding and removing these modifications or ‘marks’ are generally referred to as writers and erasers of the histone code respectively, and protein modules binding and interpreting the marks, as readers of the code [7]. While inhibitors of histone deacteylases (HDACs) have already been approved by the US FDA as drugs for a variety of cancers and HDACs are being investigated for the treatment of other pathologies [8,9] inhibitors for other epigenetic targets are only recently being explored for their therapeutic use. However, well-validated probe compounds have been made freely available for many of the epigenetic proteins with a particular good coverage of bromodomains, readers of acetylated lysines [10,11], and histone methyl transferases (HMTs), which add methyl moieties to histone tails [7].

Importantly, in order to understand the biological function of these epigenetic proteins, high-quality inhibitors are necessary. These are crucial in order to explore the role of specific domains of a protein or interrogate the catalytic versus scaffolding functions of an enzyme [12] and moreover may serve as starting points for drug discovery programs. Unfortunately, a number of inhibitors have been developed against epigenetic and other targets with poorly characterized properties. Recent publications question the quality of many of the published inhibitors, not only for epigenetic targets, and demand better characterization of tool compounds or ‘probes’ [13–15] with defined potency and selectivity criteria. The SGC chemical probe program has addressed this problem and generated greater than 30 tool compounds for epigenetic targets to date, with clearly defined properties (Box 1) [16]. An SGC chemical probe is characterized by the following properties: a potency of less than 100 nM in a biochemical or biophysical assay; selectivity of greater than 30-fold against other members of the same family; and cellular engagement of less than 1 μM. All probes are additionally profiled against a panel of pharmacologically relevant targets [17] and against a set of diverse kinases. A particular goal is to generate multiple probes from alternative scaffolds to unveil potential off-target effects.

In addition, a major hurdle for the scientific community is that many chemical probes are not generally available and are often inadequately characterized, the latter rendering correct interpretation of biological results difficult [18,19].

In contrast, inhibitors that have been made available have often had a significant impact as tools to understand the complex biology of their targets and the potential of the target in drug discovery (Figure 1). An excellent example of this are BET inhibitors, which have spurred a plethora of scientific and therapeutic advancements. Importantly, in less than 5 years after their publication, inhibitors of the BET family have entered clinical trials enabled, in part, through a solid target validation by the scientific community [20].

Here we provide a short overview about freely available chemical probes and the emerging biology based on their use by the scientific community.

## BET inhibition: a promising start

**Figure 2:**
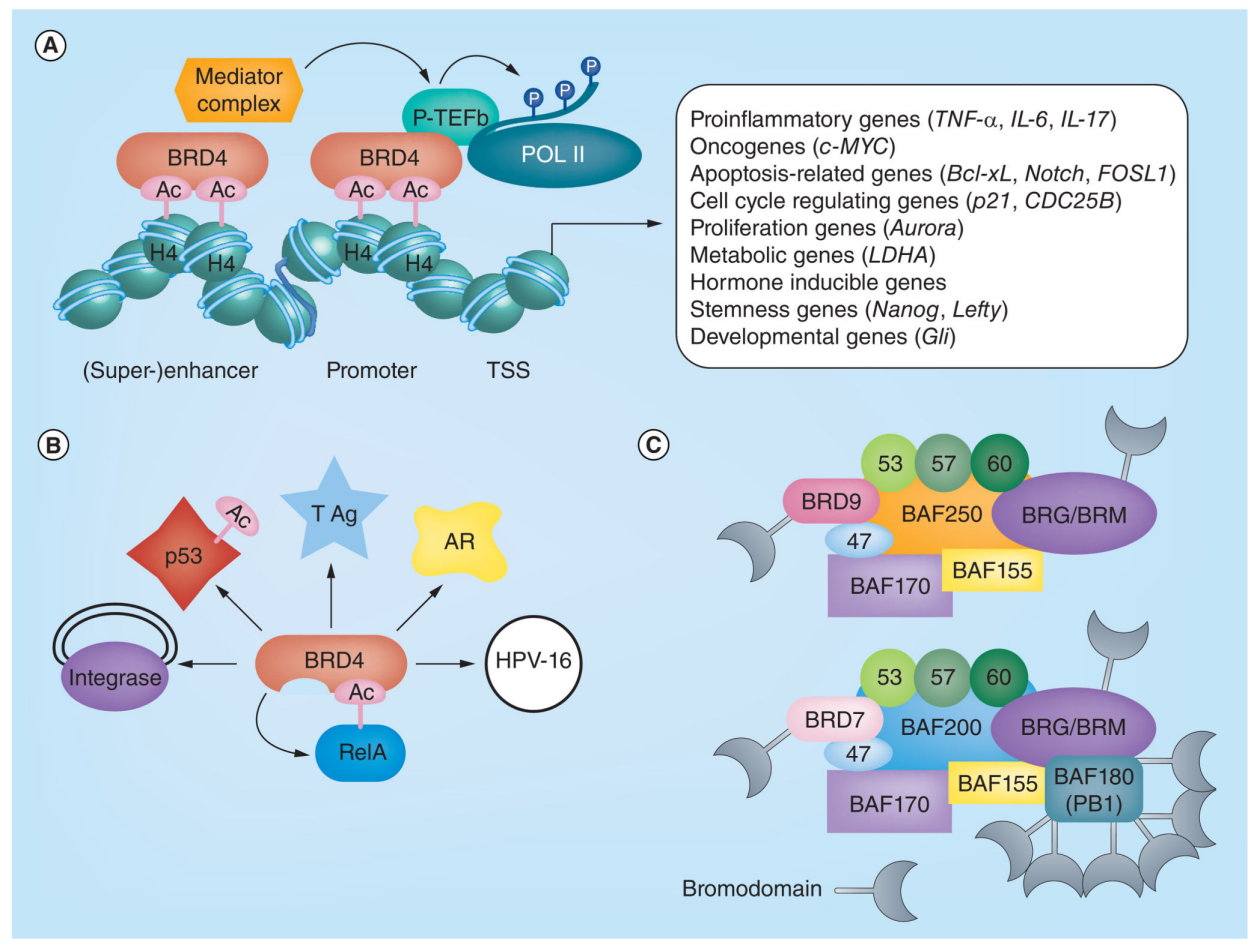
Biological effects observed using BET inhibitors and BAF and PBAF complexes **(A)** BRD4 is recruited to promoters and enhancers/super-enhancers of a variety of transcribed genes regulating the expression of key genes in a variety of biological and disease functions. **(B)** BRD4 also regulates biological functions through nonchromatin-mediated mechanisms, for example, through binding to acetylated transcription factors like RelA and p53, binding to androgen receptor (AR) or interaction with viral genes influencing viral integration or replication such as the large T antigen (TAg) of Merkel cell polyomavirus, viral factor E2, crucial for HPV16 replication (HPV-16) or retroviral integrase of murine leukemia virus (integrase). **(C)** Bromodomains as part of the BAF and PBAF complexes. 47: BAF47; 53: BAF53; 57: BAF57; 60: BAF60; Ac: Acetyl group; POL II: RNA Polymerase II; H4: Histone H4; P: Phosphorylation; TAg: Large T antigen; HPV-16: Herpesvirus 16. **(A)** Adapted from [35] © Müller *et al.* (2014).

Bromo and extra terminal (BET) family members belong to the family of bromodomain (BRD) containing proteins, which ‘read’ ε-*N*-acetylated lysine residues on histone (H3 and H4) as well as nonhistone proteins, for example, p65 subunit of NFκB. Each of the four BET proteins (BRD2, BRD3, BRD4 and BRDT) contains two conserved N-terminal bromodomains and an extra terminal (ET) protein interaction motif. Freely available BET inhibitors have greatly accelerated the knowledge of this important family of transcriptional regulators. Excellent reviews have been written summarizing the role of BET proteins in disease as well as their normal physiological functions, and we will therefore only briefly cover this crucial family of bromodomains [11,21–26]. In summary, BET proteins, and in particular BRD4, are major regulators of binding of transcription to acetylated histones and influencing transcriptional elongation. This is in part mediated by the interaction of BRD4 with the positive transcription elongation complex (P-TEFb), composed of cyclin-dependent kinase CDK9 and its activator cyclin T. Recruitment of P-TEFb to promoter regions results in phosphorylation of the C-terminal heptad repeat domain of RNA polymerase II and transcriptional elongation. In addition, BRD4 regulates gene transcription via interaction with the mediator complex from more distant transcriptional regulatory elements such as enhancers or (super)enhancers that drive the expression of key disease promoting genes regulating apoptosis, cell cycle, differentiation, inflammation or metabolism (Figure 2A). Alternatively, BRD4 acts in a chromatin-independent mechanism by directly interacting with nonhistones, either cellular transcription factor, like acetylated p65 [27], or p53 [28], the androgen receptor [29] or with viral proteins, which via interaction with BRD4, make use of the host transcriptional machinery [30] or alternatively influencing viral replication and integration (Figure 2B) [31,32]. Recently, chemically modified BET inhibitors have been used in new ways as sophisticated molecular tools. A biotinylated BET inhibitor (bio-JQ1) has been generated, which combined with parallel DNA-sequencing unravels the localization of its target proteins on chromatin. In this Chem-seq approach, the genome occupancy of the BET proteins is assessed using the modified JQ1 molecule [33]. Linking a phthalimide moiety to JQ1 (dBET1) ‘marks’ the target protein for proteolytic degradation via the ubiquitin pathway and not only inhibits BET proteins from binding to chromatin, but completely abolishes the target [34]. This strategy may be useful for other bromodomain containing proteins, in particular those, where targeting of the bromodomain has not led to a global displacement from chromatin like in the case of CBP (see below) making use of the good druggability of the bromodomain to target the whole protein including less druggable domains like the HAT domain.

Several probes from different scaffolds have been reported and we point to excellent reviews reporting on these molecules [11,36–38]. BET inhibitors have proven remarkably effective in a variety of diverse tumor types (see reviews above) and have also shown promise in the treatment of inflammatory diseases. However, a recent finding of BRD4 regulating developmental genes in the hedgehog signaling pathway may limit the use of BET inhibitors to oncology, or short-term treatments. Treatment of zebrafish with high doses of the BET inhibitor JQ1 also resulted in anomalies in line with those of observed in *Brd4* heterozygous mice [39].

## Bromodomain inhibitors beyond BET

**Figure 3:**
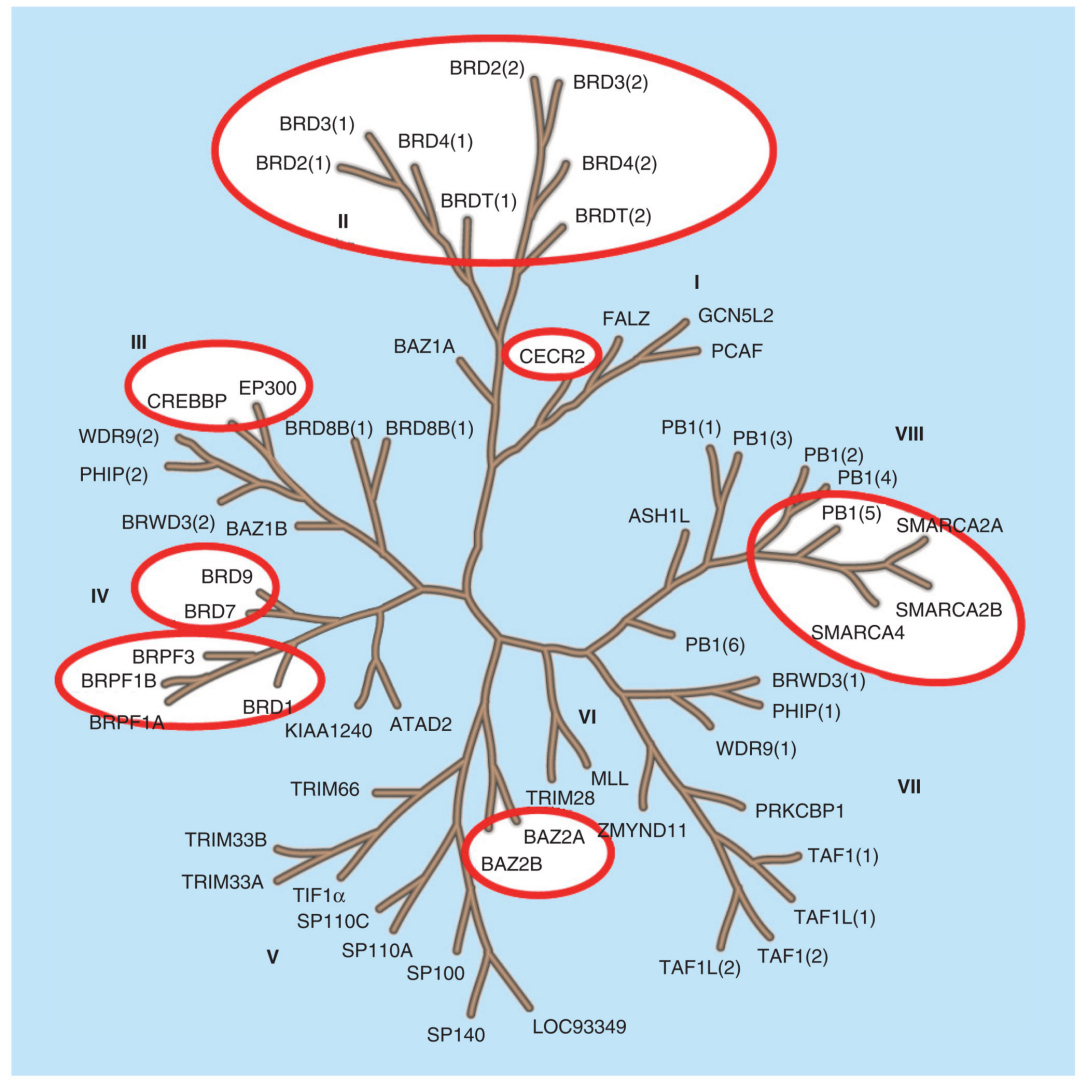
Bromodomain tree and available probe molecules Circled are bromodomain targets for which chemical probes have been generated. Tree adapted according to Filippakopoulos *et al.* [10].

**Figure 4:**
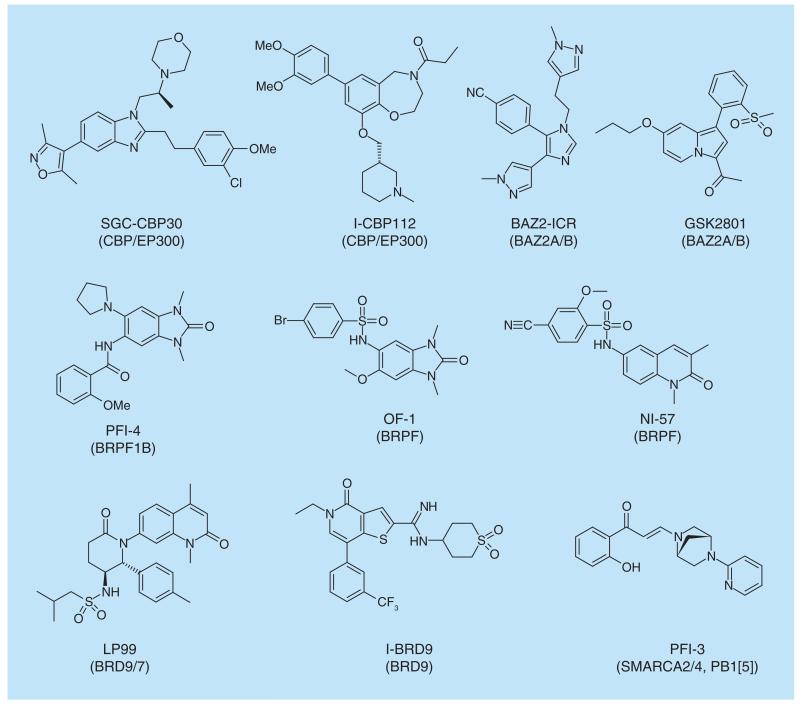
Bromodomain inhibitors for non-BET bromodomains.

Although most of the efforts have been concentrated on BET inhibition, chemical probes for many of the bromodomain branches have been generated (Figures 3 & 4).

Several different inhibitors for the CREB binding protein, CBP, and E1A binding protein, EP300, have been described. CBP and EP300 are general transcriptional co-activators, which possess a catalytic histone acetyltransferase (HAT) domain in addition to the bromodomain, which catalyzes the transfer of acetyl moieties to lysine on histone, in particular H3K56, and nonhistone proteins. These proteins play central roles in many biological processes including cell growth, genomic stability, development, neuronal plasticity and memory formation as well as energy homeostasis [40]. Although the CBP bromodomain inhibitors do not globally displace exogenously expressed full-length CBP from chromatin [41,42] inhibition of the bromodomain has an effect on H3K56 acetylation levels implying that the CBP bromodomain promotes acetylation of H3K56 by mechanisms dependent on the interaction between acetylated lysine and the bromodomain [43]. Both CBP and EP300 proteins have been shown to be essential players in leukemogenesis [44], but little is as yet known about the role of the bromodomain in these processes. CBP inhibitors from two different chemical scaffolds against the bromodomain have been generated, the oxoazepine, I-CBP112 and the slightly more potent isoxazole, SGC-CBP-30. Both probes fulfill criteria set out in Box 1, but have weak off-target activity for BRD4 and other BET family members. Thus, using these probes at high concentration (>>2 μM) should be avoided. Considering the effect of BET proteins in a plethora of transcriptional physiological and pathophysiological processes, it is no surprise that at higher concentrations of both CBP inhibitors, a BET effect is the dominant fingerprint of the compound [45,46; Picaud *et al.*, Unpublished Data] . Among the CBP/p300-specific phenotypes, we found that I-CBP112, contrary to the BET inhibitor JQ1, did not show immediate cytotoxic effects on leukemic cell lines immortalized by the *MLL-CBP* fusion and other potent leukemia-associated oncogenes including the *MLL-AF9*, *MLL-ENL* or the *NUP98-HOXA9*, which were exposed to the compound, but instead impaired colony formation and induced cellular differentiation. Initial *in vitro* and *in vivo* experiments point to a potential application of CBP bromodomain inhibitors targeting leukemia stem cell renewal [45]. Interestingly, both BET and CBP cooperate on promoters in leukemia cells and acetylation of key transcription factors as well as histone tails by CBP leads to direct binding and recruitment of BRD4 to promoters in leukemic cells [47]. Indeed, applying this knowledge it may be therapeutically beneficial targeting both BET and CBP bromodomains by dual specificity inhibitors.

Several probes have been reported targeting different members of the SWI/SNF remodeling complexes BAF and PBAF. BAF and PBAF complexes are large multisubunit complexes involving several bromodomain-containing proteins including the mutually exclusive catalytically active components SMARCA2/BRM and SMARCA4/BRG1. BRD9 is associated with the BAF complex, whereas BRD7 is part of the PBAF complex, which also harbors 6-bromodomain containing protein polybromo 1 (PB1 or PBRM1) anchoring the complex to chromatin (Figure 2C) [48,49]. Mutation of components of the BAF complex have been found in 20% of all cancers resulting typically in loss of function pointing to tumor suppressor roles of the BAF complex [50]. For example, mutations of *PB1* are frequently found in renal cell carcinoma and expression levels of PB1 are a prognostic marker for metastatic renal cell carcinoma [51,52] and the SWI/SNF subunit BRG1 has been shown to be required for maintenance of acute myeloid leukemia [53].

The related proteins BRD9 and BRD7, form a small subbranch in the bromodomain tree containing each a single bromodomain. Little is known about the functional and pathophysiological role of BRD9, but the protein has been found to be overexpressed in cervical cancer due to a gain of the short arm of chromosome 5 (5p) [54]. BRD7 on the other hand, is frequently down-regulated in cancer and has a proposed tumor suppression function through regulation of PI3K [55] and p53 [56,57]. Knockout studies of *Brd7* revealed that Brd7 is necessary for cognitive behavior and Brd7 knockout mice show reduced synaptic-related proteins expression and dendritic spines or branching in the medial prefrontal cortex. In good agreement with the observed phenotype, Brd7 was found to be expressed in neurons but not glial cells.

Three probes from different scaffolds have been reported targeting the BRD9/7 family [56,58–59] LP99, and BI-9564 inhibit both BRD9 and BRD7, but with a preference for BRD9. LP-99 binds to BRD9 with a Kd of 99 nM and to BRD7 with a Kd of 909 nM. BI-9564 is about sevenfold more potent on BRD9 (Kd = 14 nM) and fourfold more potent on BRD7 (Kd = 239 nM). These inhibitors are complemented by a BRD9 selective compound I-BRD9 [59]. Although few phenotypic effects have been reported using these compounds apart from their cellular target engagements, initial data point to a role of BRD9/7 in inflammation. So far there have been no reports in the literature linking BR9 or BRD9 to inflammatory diseases, but chromatin remodeling by the BAF complex has been shown to be important for the transcriptional expression of inflammatory genes [60]. Using LP99, a reduction in IL-6 release of LPS stimulated THP-1 cells has been demonstrated [56]. The availability of three well- characterized probes will easily lend credibility to these observations or disprove the initial observations.

A probe (PFI-3) has also been described targeting the bromodomains of the catalytic SWI-SNF subunits BRG1 and BRM. In addition, the compound also binds tightly to the fifth bromodomain of the BAF factor PBRM1 (PB1(5)). Given the central role of SWI/SNF in cancer [53] the compound was tested in several different cancer cell lines. However, the initial screening in cancer cell lines revealed no antiproliferative effect of the compound PFI-3 [61,62], indicating that cell proliferation may not be mediated by targeting the bromodomain. Indeed, in hematopoietic malignancies it has been demonstrated that the ATPase domain and not the BRD is essential for cell proliferation and survival [26]. Instead a crucial role for the BRG/BRM BRDs during early differentiation influencing stem cells was identified using PFI-3 [62]. This may point toward a useful role of PFI-3 targeting cancer stem cells, but also highlights potential dangers to use this and related compounds also outside the oncology area.

Specific chemical probes have also been described for the BRomodomainand PHD Finger containing (BRPF) family of bromodomains (Figure 4). This family comprises three family members BRPF1, BRPF2 (alias BRD1) and BRPF3. BRPFs are scaffolding proteins for HAT complexes of the MYST family (MOZ, Ybf2/Sas3, Sas2 and Tip60). MYST complexes have a tetrameric core composed of BRPF, the tumor suppressor ING and Eaf6/EPC (enhancer of polycomb) related scaffold subunits. They play a role in a variety of key nuclear processes and play critical roles in gene-specific transcription regulation, DNA replication as well as DNA damage response and repair. MOZ is frequently translocated in leukemia and it has been shown that this HAT is required for hematopoietic stem cell proliferation [63]. MOZ together with MLL regulates *HOX* transcription factors in haematopoiesis, and deregulation of these HATs could further contribute to leukemogenesis [64]. Through the regulation of *HOX* genes, MOZ also influences ES cells with respect to body segment identity specification [65] and similar roles in development through *HOX* gene regulation have been shown for BRPF1 in zebra fish [66]. Accordingly, *Brpf1* knockout causes embryonic lethality at E9.5. Mutant embryos show vascular defects in the placenta, yolk sac and embryo proper as well as abnormal neural tube closure demonstrating the crucial role for Brpf1 in embryo development [67] The related BRPF2 is also critical for hematopoietic lineage commitment [68,69]. Several probes have been developed for targeting either all family members [70,71] or only BRPF1B [72], but their biological effects have not been published yet. Interestingly, two dual-specificity inhibitors have recently been published targeting BRPF1 in addition to the potential breast cancer target TRIM24 [73,74]. Characterization of these and the more specific compounds will help validating these targets in leukemia and other oncological diseases.

## Emerging target areas: KDMs

**Figure 5:**
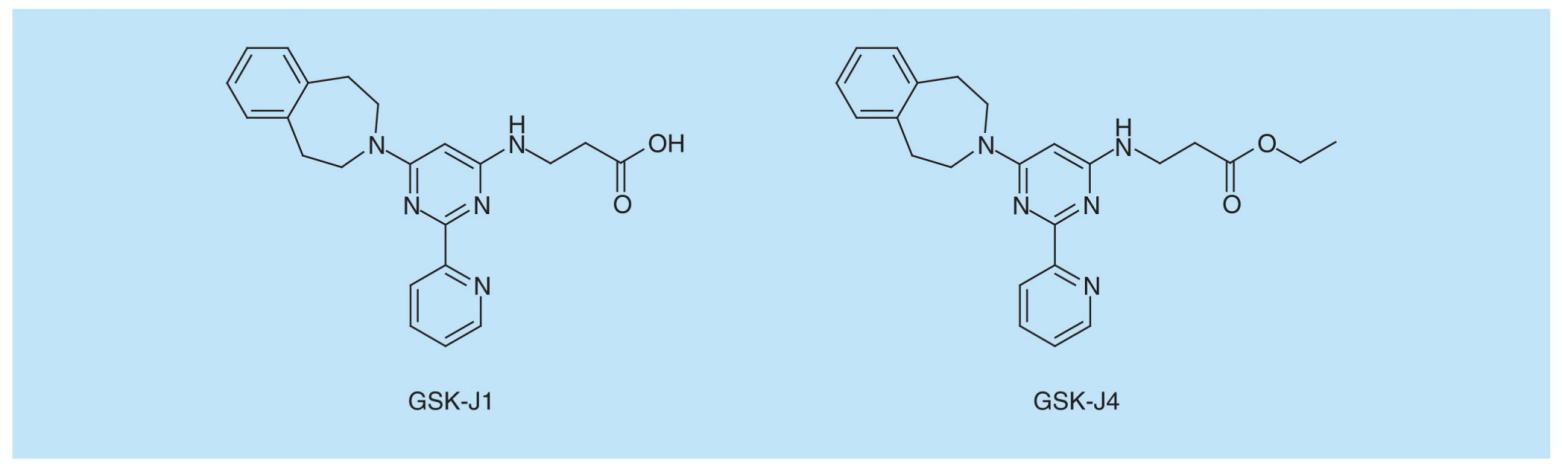
Chemical structures of the KDM6 inhibitors GSK-J1 and the ester prodrug GSK-J4.

No true lysine demethylase probe has been described yet, but GSK-J1 (Figure 5) is a potent KDM6 inhibitor inhibiting both KDM6A and KDM6B, which are 2-oxoglutarate and Fe^2+^ dependent oxygenases. However, it does not quite fulfill selectivity criteria as it only has a 15-fold selectivity over another histone lysine demethylase JARD1B [75,76]. Due to its carboxyl group, GSK-J1 does not have potent cellular activity, but the ethyl ester prodrug, GSK-J4 (Figure 5), has been shown to be an effective cellular probe. Using this compound several therapeutic starting points have been revealed. KDM6A and KDM6B catalyze the removal of the repressive mark trimethylated lysine 27 of histone H3 (H3K27me3). KDM6B plays a crucial role in development but has also been implicated in several diseases, notably cancer and inflammation [77]. The proposed role for KDM6B in inflammation has been confirmed by the use of GSK-J4, which reduces production of the pro-inflammatory cytokine TNF-α from macrophages derived from patients with rheumatoid arthritis [75]. GSK-J4 also has been shown to have potential in autoimmune responses as it suppresses differentiation of T helper 17 (Th17) cells by increasing the repressive H3K27 trimethyl (me3) mark at the genomic sites of the key transcription factor for Th17 cells, Rorc as well as Th17 cytokine genes such as *IL17*, *IL17f* and *IL22* [78]. Both KDM6B and KDM6A (UTX) have been shown to be crucial for the reactivation of herpes simplex virus 1 (HSV-1). During the latent phase of the HSV-1 life cycle, the integrated virus genome is associated with repressive marks including H3K27me3 along lytic genes keeping them in a repressed state. GSK-J4 prevents removal of the H3K27me3 mark and therefore reactivation of HSV from latency providing a potential therapeutic avenue for recurrent HSV disease [79].

Also in oncology, GSK-J4 has been shown to have therapeutic potential. The compound was effective in inhibiting growth of T-cell acute lymphoblastic leukemia (T-ALL) cells and the gene expression signature observed using the inhibitor resembled that of shJMJD3 implying that the observed effect is to a large extent mediated by inhibition of KDM6B [80]. Also in glioma, GSK-J4 has shown a strong antitumor activity both *in vitro* and in a mouse xenograft model. The antiproliferative effect in the K27M tumor cells was accompanied by an increase in cellular H3K27 methylation [81]. Combination studies with the pan-HDAC inhibitor panobinostat and GSK-J4 moreover demonstrated a synergistic effect in patient-derived diffuse intrinsic pontine glioma cultures [82].

## Protein methyltransferases

**Figure 6:**
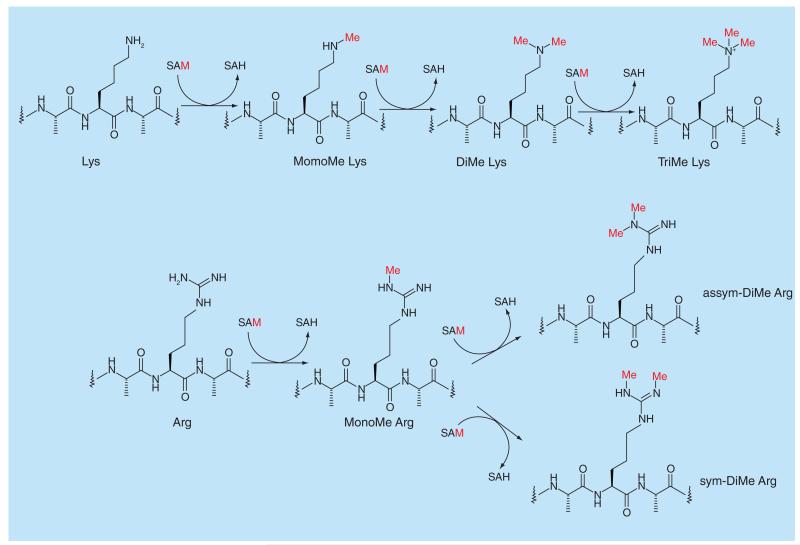
Methylation of lysine and arginine by *S*-adenosyl methionine.

**Figure 7:**
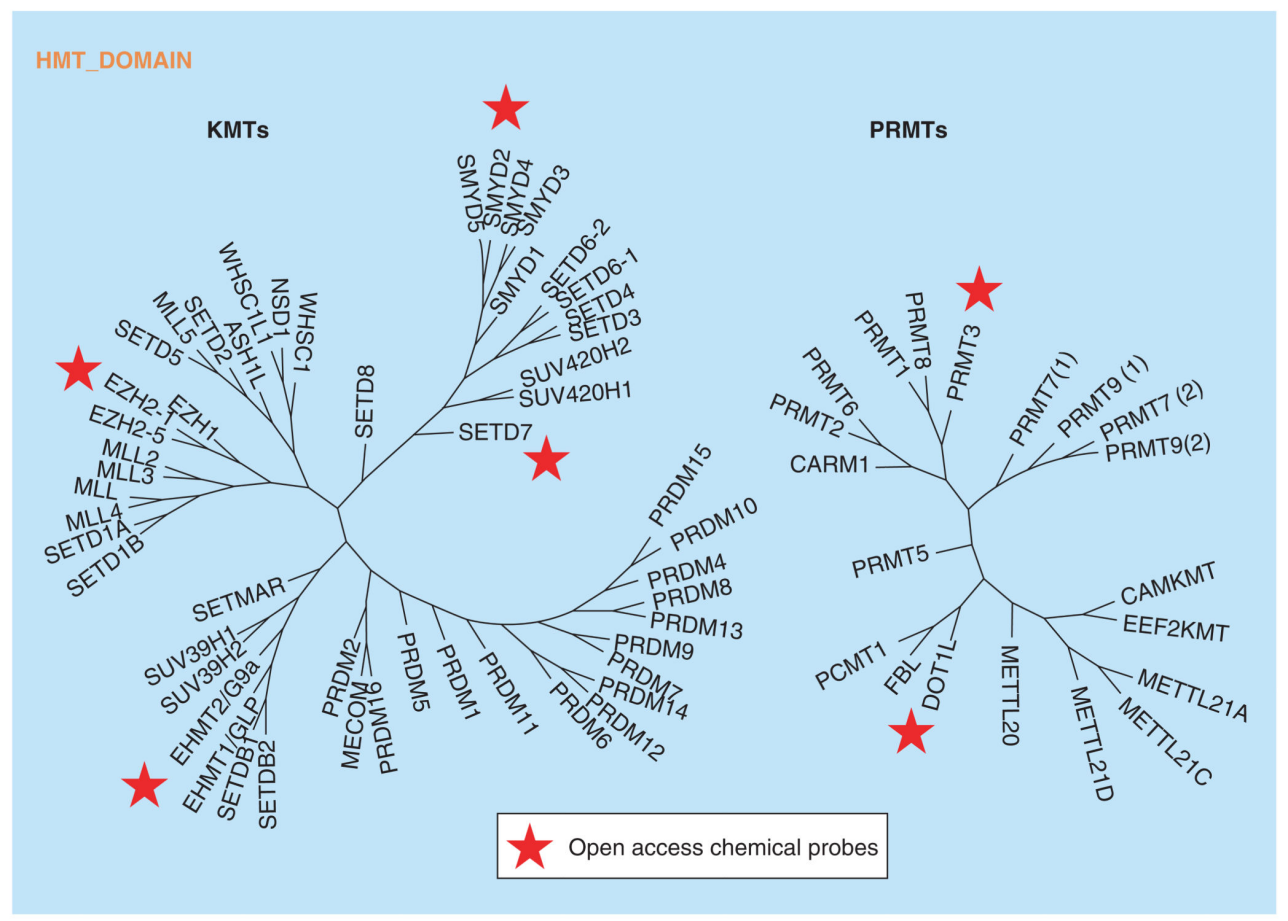
Phylogenetic tree of histone methyl transferases and arginine methyl transferases with generated chemical probes Targets for which an open access chemical probe has been generated are marked by a star.

Protein methyltransferases (PMTs) mono-, di- or tri-methylate-specific lysine residues, or mono- or dimethylate arginine in histone and nonhistone substrates using S-adenosyl methionine (SAM) as cofactor. The PMTs are very selective in the position of the lysine which is targeted for methylation and the extent of lysine methylation is controlled by the size of the lysine-binding pocket. Some lysines can be mono-, diand tri-methylated by the same protein while others need the sequential action of multiple proteins for full methylation. Similarly for arginine PMTs, selectivity if fairly high and in addition the dimethylated products are either symmetric or asymmetric (Figure 6). Several probe molecules have been generated for PMTs from different branches of the phylogenetic tree (Figure 7).

Most of the lysine PMTs contain a SET domain which is responsible for the catalytic activity. This domain is named after the three *Drosophila* genes in which it was originally identified (**S**uvar(3–9), **E**nhancer of zeste and **T**rithorax), the exception being DOT1L which contains a Rossman fold, similar to all the other arginine PMTs. Thus DOT1L resides in the PRMT branch of the PMTs.

**Figure 8:**
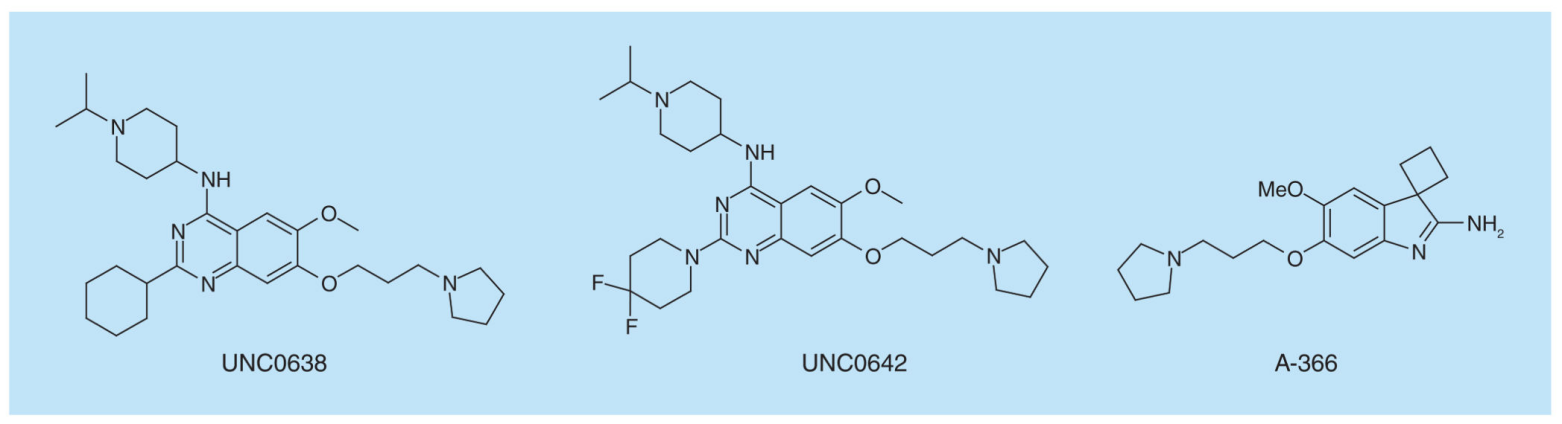
G9a/GLP inhibitors.

G9a (EHMT2) and a closely related protein GLP (EHMT1) catalyze the mono- and di-methylation of histone 3 lysine 9 (H3K9). The first potent inhibitor of a protein methyltransferase is BIX-01294 which was published in 2007 exhibited low microM potency on both G9a (EHMT2) and GLP (EHMT1), and showed some cellular toxicity at higher doses [83]. This was used as a template for further analogs by Jin, resulting in the more potent probe, UNC0638 (Figure 8), which exhibited lower toxicity at higher doses [84]. A further analog, UNC0642 (Figure 8), was developed to enable the inhibition of G9a/GLP to be studied in animal models, including those which required some brain penetration [85]. These two probes for G9a/GLP have been used in several studies in order to delineate the role of these protein methyltransferases in biological processes and potentially identify those in which inhibitors could be of use clinically. Sharp-1 has been shown to modulate the differentiation of myoblasts into muscle cells, probably by recruitment of G9a. Studies have shown that both knockdown of *G9a* and inhibition of G9a activity using UNC0638 eliminate Sharp-1-mediated differentiation [86]. In addition, studies on PRDM16-mediated repression of myogenesis show that knockdown of *G9a* or inhibition of G9a activity with UNC0638, eliminates the repressive effects of PRDM16 on myogenesis [87]. Further studies in both murine and human AML cells indicate that inhibition of G9a by UNC0638 selectively affects the proliferation of AML cells without affecting stem cells [88]. A chemical probe for G9a/GLP from a different chemical template, A-366 (Figure 8), shows significantly lower cellular toxicity [89].

**Figure 9:**
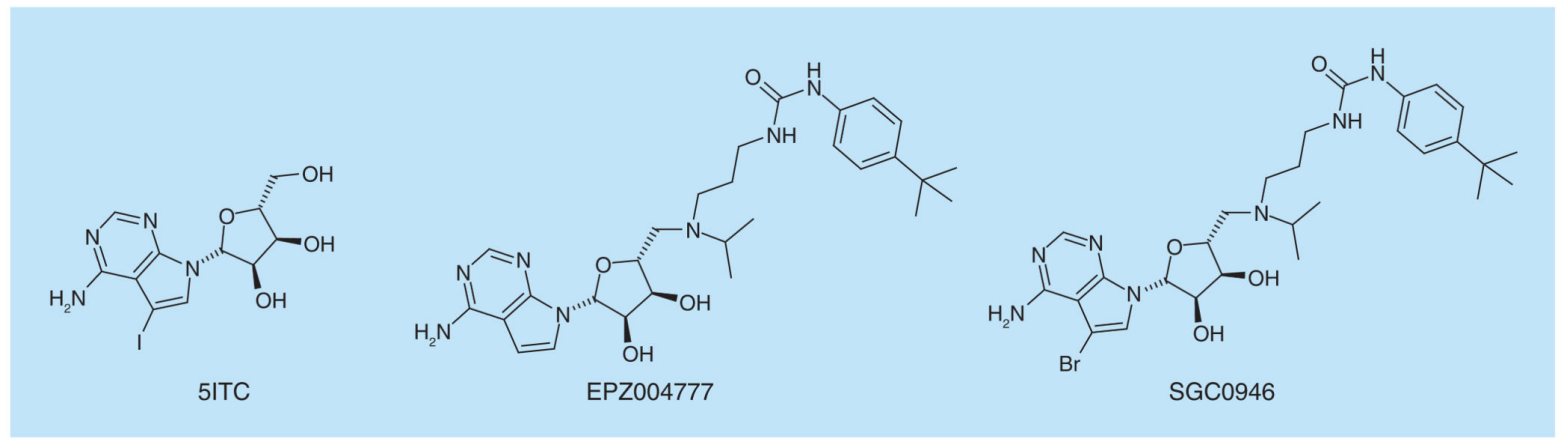
DOT1L inhibitors.

DOT1L catalyzes the mono- and di-methylation of histone 3 lysine 79 (H3K79) and has been implicated in the pathogenesis of MLL. This protein is not active on histones unless they are incorporated into a nucleosome structure. The rate of H3K79 methylation is greatly enhanced (44-fold) when the H2B portion of the nucleosome is ubiquitinylated at K120 [90]. Early work on identifying inhibitors of protein methyltransferases involved screening of libraries of kinase inhibitors as many of these are analogs of ATP, a close mimic of S-adenosylmethionine, the methyl-donor cofactor in protein methylation reactions. 5-iodotubercidin (5ITC) was identified as an 18 μM inhibitor of DOT1L via this mechanism and was crystallized with DOT1L showing that this ligand bound in the SAM-binding pocket of DOT1L (Figure 9) [91]. Growing this molecule to contain the SAH amino acid side-chain and replacing the iodo group with a chemically more feasible bromo group afforded bromo-SAH (IC_50_= 77 nM). This halogen group occupies a pocket in the SAM-binding site which is unique to DOT1L and is responsible for the high selectivity of bromo-SAH over other methyl transferases. Concomitantly, Epizyme published [92,93] a manuscript describing EPZ004777 (Figure 9) which incorporates most of the SAM structure, except the polar amino acid was replaced with a lipophilic t-butylphenylurea moiety. The publication of this highly potent molecule also elegantly showed how inhibition of DOT1L results in the selective death of cells containing the *MLL* gene translocation. Incorporation of the tail of EPZ004777 into the bromo-SAH molecule resulted in SGC0946 (Figure 9) with potent DOT1L inhibitory properties *in vitro* and in cellular setting [94]. As a result of the selective killing of MLL rearranged cells, several companies have DOT1L inhibitors in the clinic for treatment of leukemia.

**Figure 10:**
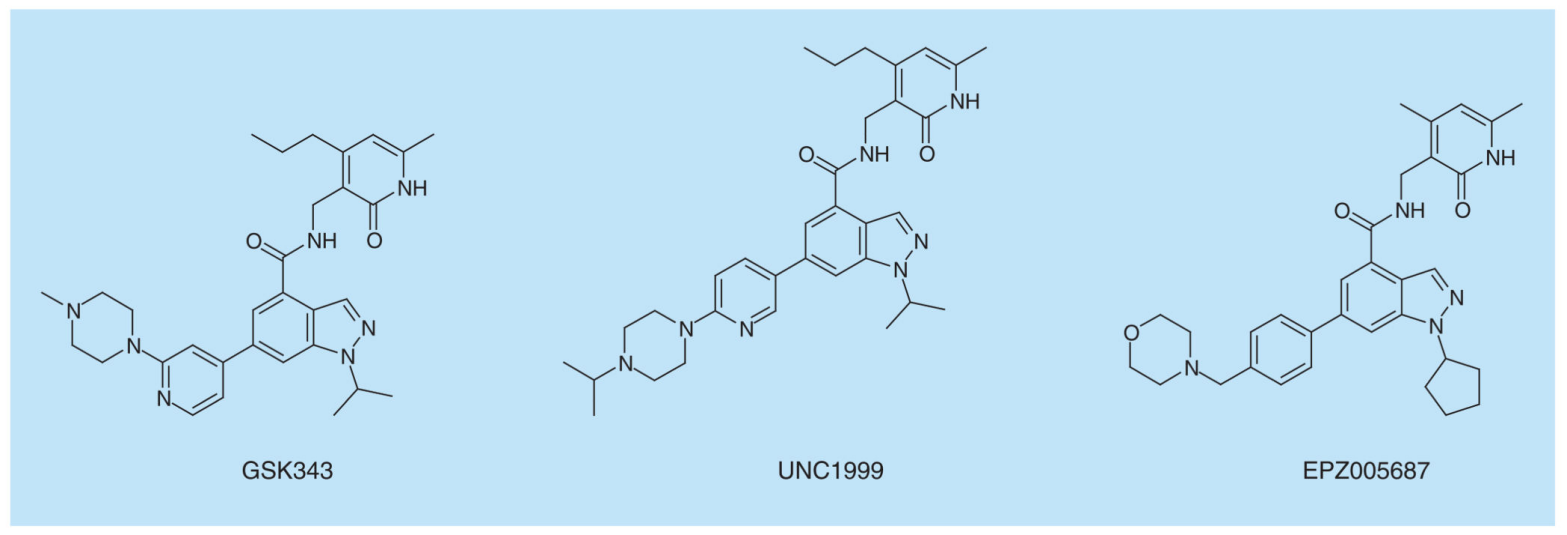
EZH2 inhibitors,

EZH2 is a component of the PRC2 complex which catalyzes the mono-, di- and tri-methylation of histone 3 lysine 27 (H3K27). Overexpression of EZH2 and high levels of H3K27me3, are hallmarks of several cancers, making this a prime oncology target. The high level of H3K27me3 in tumors correlates with the presence of EZH2 mutants at position Y641 which have been shown to have increased K_cat_ for H3K27me2 over that for the wild-type [95]. However, while the K_cat_ for H3K27me2 as substrate is increased, the K_cat_ for the corresponding H3K27 and H3K27me substrates is greatly reduced compared with wild-type. This leads to an intriguing situation in which the presence of both wt and mutant EZH2 is needed for tumor progression. As in the case of DOT1L, screening libraries of kinase inhibitors afforded starting points for chemical optimization leading to chemical probes GSK343 [96] and EPZ005687 (Figure 10) [97]. Further optimization gave UNC1999, which has improved pharmacokinetic properties and is suitable for *in vivo* use [98]. Treatment of a diffuse large B-cell lymphoma (DLBCL) cell line which contains the Y641N mutant EZH2 with UNC1999 (Figure 10) for 8 days resulted in a potent antiproliferative effect, which is not observed following treatment with UNC2400, a related molecule which is much less active as an EZH2 inhibitor. In the light of this data, EZH2 inhibitors have entered clinical trials.

**Figure 11:**
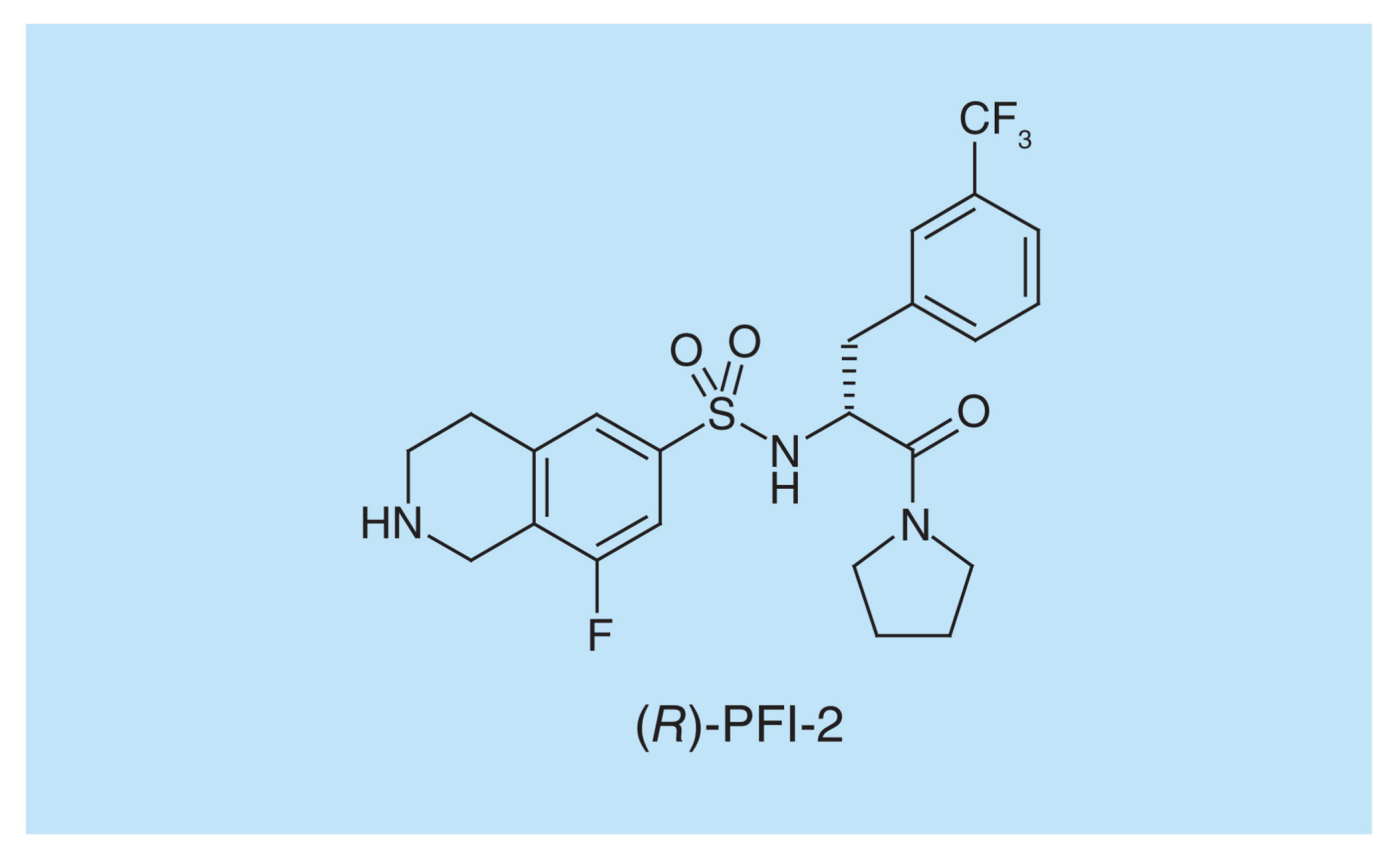
SETD7 inhibitor.

SETD7 catalyzes the methylation of histone 3 lysine 4 (H3K4) *in vitro* but has been shown to methylate multiple substrates in cells [99–101]. *Setd7* knockout mice do not exhibit any noticeable phenotype and thus any effects from inhibiting this protein might be subtle. *(R)*-PFI-2 (Figure 11), a potent and selective inhibitor of SETD7 [102], was developed from an early hit from high-throughput screening, and was shown to affect the Hippo pathway by regulating the localization of YAP from the cytosol to the nucleus. The *(S)*-enantiomer is an effective control for this experiment, showing significantly lower inhibitory activity (500-fold lower). While the standard kinetic analysis of SETD7 inhibition by *(R)*-PFI-2, seems mixed (as opposed to SAM-competitive or peptide competitive), it is evident from SPR studies that *(R)*-PFI-2 binding is SAM-dependent. In this manner, classical peptide-competitive behavior is only observed at SAM concentrations below its K_m_.

**Figure 12:**
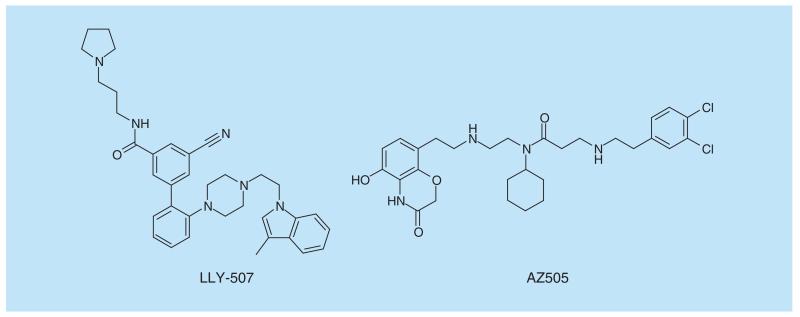
SMYD2 inhibitors.

SMYD2 is a PMT which methylates histone 3 lysine 36 (H3K36) and K370 of p53 among other proteins [103–105]. SMYD2 is implicated in the development of breast and liver cancers which have been shown to exhibit gene amplification and/or overexpression of SMYD2. A few potent inhibitors have been discovered for SMYD2, notably, AZ505 [106], which has limited cellular activity, and more recently, LLY-507 (Figure 12), which is a cellularly active peptide-competitive inhibitor [107]. LLY-507 potently inhibited the proliferation of several ESCC, HCC and breast cancer cell lines after dosing for 3–7 days.

**Figure 13:**
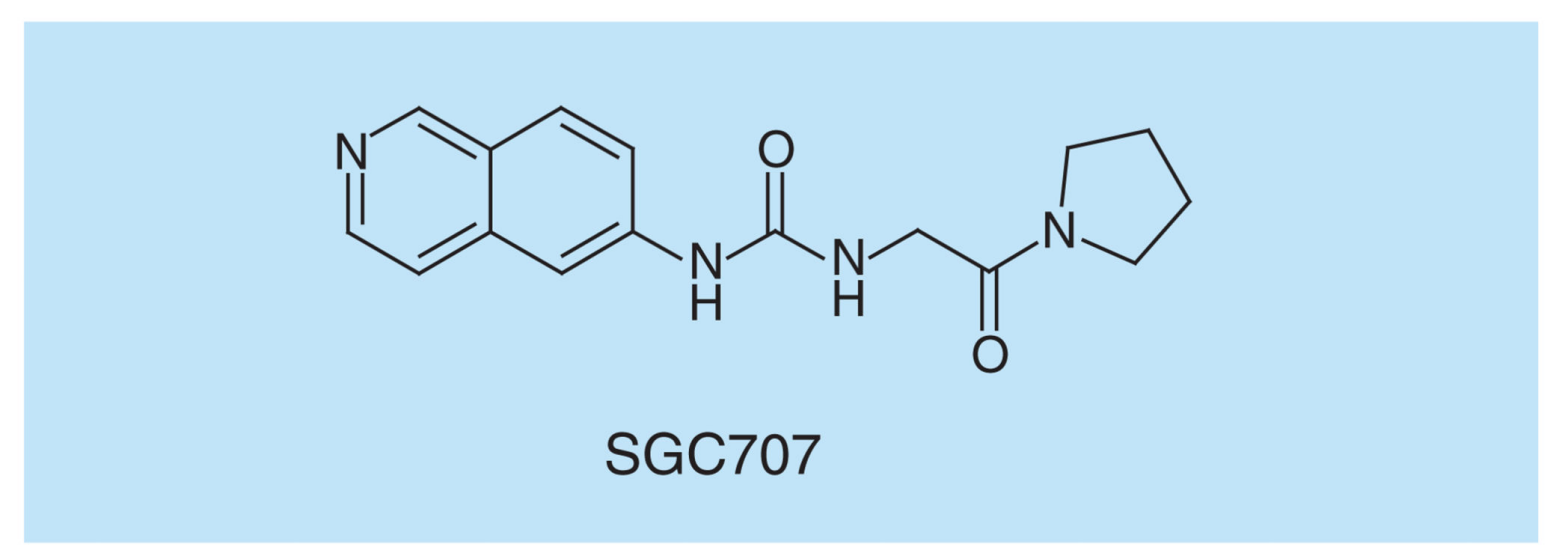
PRMT3 inhibitor.

PRMT3 is a member of the Protein Arginine Methyl Transferase family of which nine members are currently known. PRMT3 is a type I PRMT, which mono- and asymmetrically dimethylates arginine residues (Figure 6). SGC707 (Figure 13) is a potent, selective allosteric PRMT3 inhibitor, being noncompetitive with both SAM and peptide substrate [108]. The allosteric binding of SGC707 (Figure 6) to PRMT3 was confirmed by X-ray crystallography and mutation of key residues in PRMT3.

## Methyllysine/arginine binders

This family of proteins contains about 250 members which bind different methylation states of lysine and arginine. Potent small molecule antagonists of methyl lysine binders are rare and this is an area of opportunity for small molecule discovery.

**Figure 14:**
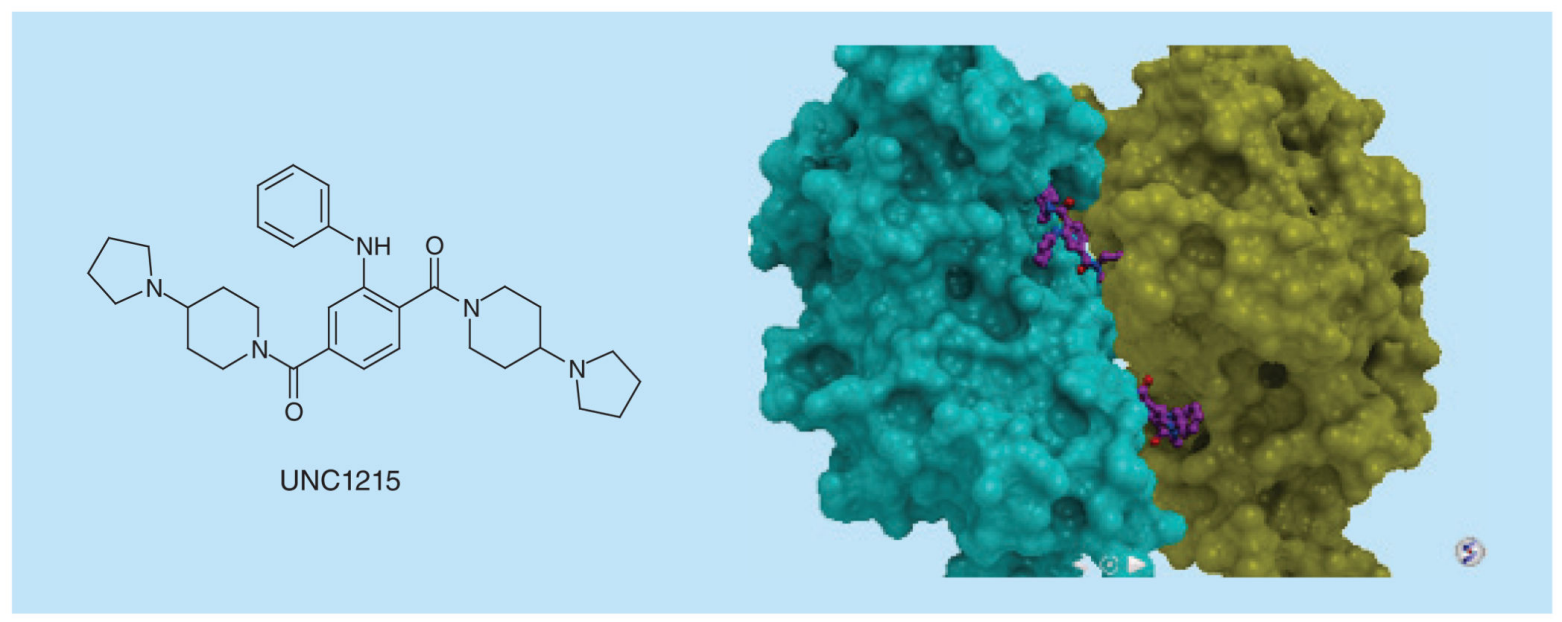
L3MBTL3 antagonist and crystal structure.

The first potent probe for one of these members is UNC1215 which was shown to be a potent, selective antagonist of L3MBTL3 [109]. This protein binds mono- and di-methylated H4K20 and contains three MBT domains which act in concert to interact with methylated H4K20 (isolated MBT domains do not bind peptides, unlike bromodomains). The crystal structure of UNC1215 with L3MBTL3 was found to be a 2:2 dimer in which each ‘arm’ of UNC1215 bound to a different protein molecule. This stoichiometry was confirmed by gel filtration studies showing dimerization upon UNC1215 binding (Figure 14).

**Figure 15:**
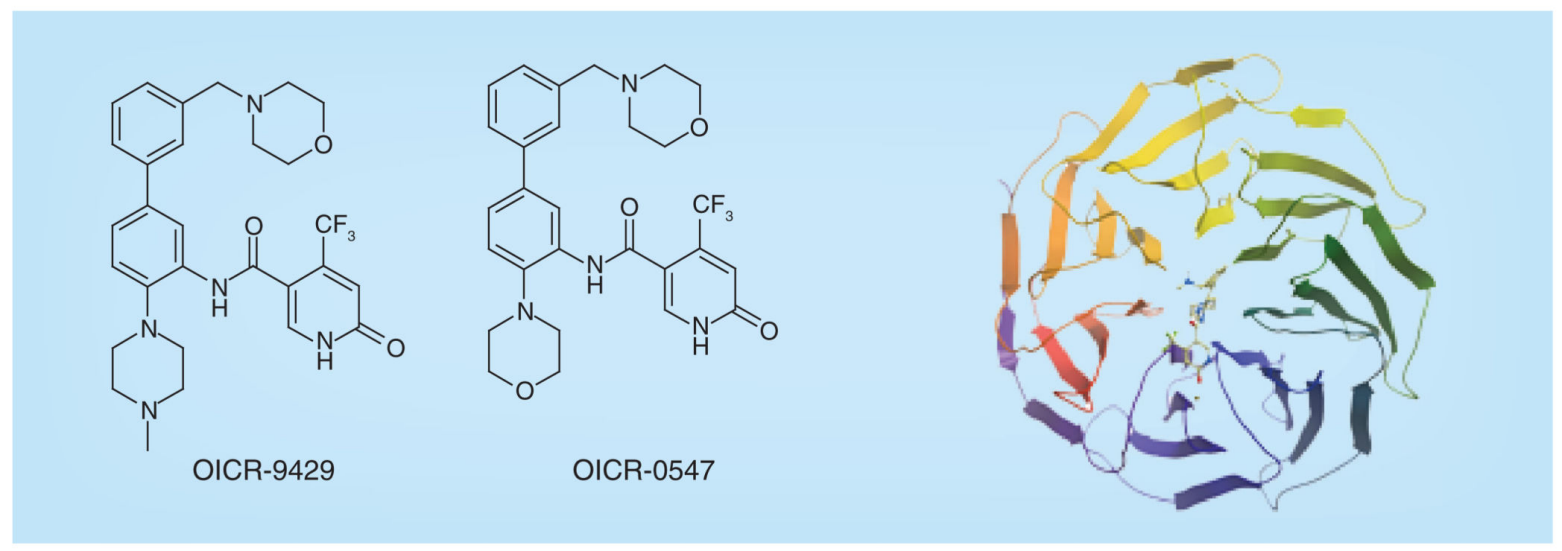
WDR5 antagonist and crystal structure.

WDR5 is a methyl arginine binding protein which is part of the MLL complex that methylates histone 3 lysine 4 (H3K4). The other components of this complex are RbBP5 and ASH1L. WDR5 contains seven propeller vanes and is readily crystallized with small molecule ligands. A weak antagonist of WDR5 binding to WIN-peptide was identified [110] using a fluorescence polarization assay (FP) through screening of a 20,000 diverse compound set and this was optimized to give OICR-9429 and an inactive control, OICR-0547 (Figure 15) [111]. The crystal structure of OICR-9429 bound to WDR5 shows that the methylpiperazine moiety binds in the middle of the seven-membered propeller structure (PDB: 4QL1). A biotinylated analog of OICR-9429 was shown to pull-down WDR5 from cell lysates and this pull-down was inhibited by unlabeled OICR-9429. In other experiments, immunoprecipitation with WDR5 and blotting for MLL and RbBP5 indicated that the MLL complex is disrupted by increasing concentrations of OICR-9429. Furthermore, OICR-9429 was shown to selectively inhibit proliferation and induce differentiation in human p30-expressing AML cells.

## Conclusion

Well-characterized freely available tool compounds are crucial to open up new areas of biology for specific targets. They often provide the first step for therapeutic intervention and are instrumental for target validation. New tools are particularly important for underexplored epigenetic targets and to elucidate the role of a specific domain in a target identified by knockdown studies. Early successes of open access inhibitors have been driven by Bromodomain and, in particular, BET inhibitors, but as more probes become available insights into other target areas and therapeutic potentials are emerging. While the search for ligands for some target classes of the epigenetic targets is difficult because of low hit rates from high- and medium-throughput screening, often, weak hits can be optimized to potent, selective probe molecules. The open availability of these probes enables the rapid advancement of knowledge about the biology of these targets and their therapeutic potential. The syntheses of these probes are shared with three major commercial suppliers of small molecules to the medicinal chemistry community and in the last 4 years, over 6000 samples of SGC probes have been used in various experiments proving the validity of the open access approach.

## Future perspective

While open access chemistry was met with a lot of skepticism 5 years ago, the success of freely available probes in accelerating target validation and discovery of new biology has spurred many open access programs in the pharmaceutical industry. It is hoped that the boundary between open access and proprietary research can be pushed further and that clinical studies can be performed in the open. High validated tool compounds will become standard and databases with chemical probes will be freely available to the community to accelerate high-quality research and drug discovery. This hopefully will have an impact on the attrition rate of compounds failing in clinical trials for the benefit of all.

## Figures and Tables

**Figure 1:**
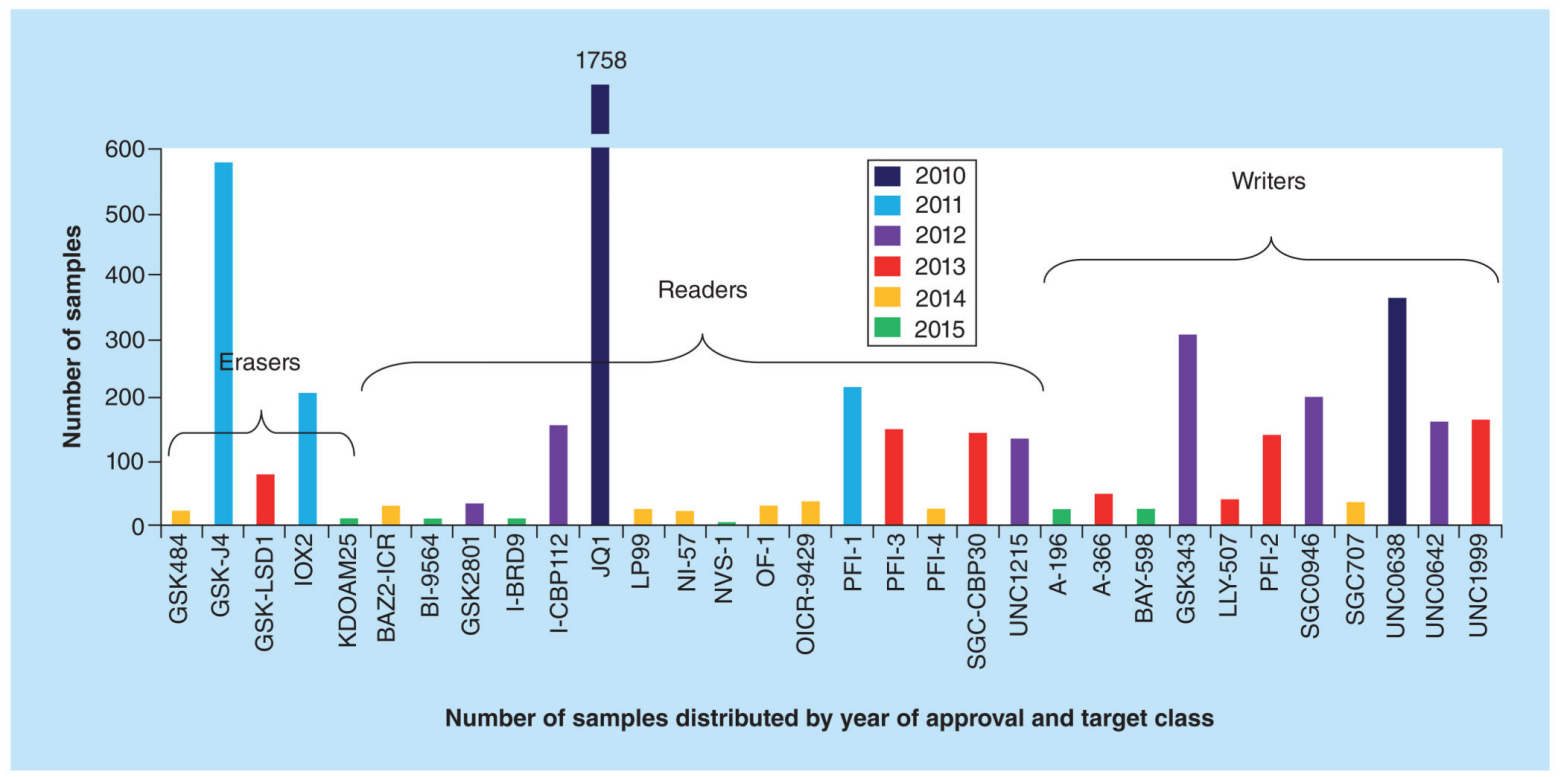
SGC epigenetic probe distribution by year of approval and target class SGC probes meet the criteria outlined in the text and are reviewed by a Joint Management Committee (Consortium partners) and an External Scientific Committee (Academics not part of Consortium).

## Key terms

### Epigenetics

Heritable changes in gene expression or cellular phenotype without alterations in the DNA sequence; epigenetic modifications include post-translational modifications of the DNA and histone tails as well as RNA transcripts and microRNAs.

### Bromodomains

Protein domains binding to acetylated lysine residues (acetyl lysine reader domains).

### Methyl transferases

Enzymes adding methyl moieties to lysine residues (methyl lysine writers).

### Chemical probes

Potent, selective cell-permeable and well-characterized small molecule inhibitors.

## Box 1 Probe criteria for chemical probes

- Potency (K_i_, K_d_, ICso): <100 nM *in vitro*
- Selectivity over other subfamilies within protein family: >30-fold
- Cell activity on target in intact cell: <1 μM

## Key term

### Lysine demethylases

Enzymes removing methyl moieties from lysine residues (methyl lysine erasers).

## Key terms

### Methyl lysine binder

Protein domains binding to methylated lysine residues (methyl lysine reader domains).

### Open access

Freely available to everyone without restriction, for example, open access chemical probes are available without restrictions on use and have greatly accelerated research on several target proteins.

## Executive summary

- Epigenetics is a new exciting area for therapeutic intervention. The area has gained momentum by the availability of highly validated chemical tool compound ‘probes’.
- Bromodomain inhibitors for the BET family have entered clinical trials in less than 5 years after the first publication.
- There is a high demand for freely available compounds in the scientific community shown by the uptake of these probes.
- Bromodomains have been successfully targeted and probes are available for the majority of the branches of the phylogenetic tree.
- Methyltransferases are the other epigenetic family for which probe generation has been successful and has opened up new areas of therapeutic intervention.
- Few lysine demethylase inhibitors are available, but with the available tool compound therapeutic potential in oncology and inflammation has been uncovered.
- A largely underexplored area is the large number of methyl lysine reader domains.
